# The Coexistence of Asthma and Chronic Obstructive Pulmonary Disease (COPD): Prevalence and Risk Factors in Young, Middle-aged and Elderly People from the General Population

**DOI:** 10.1371/journal.pone.0062985

**Published:** 2013-05-10

**Authors:** Roberto de Marco, Giancarlo Pesce, Alessandro Marcon, Simone Accordini, Leonardo Antonicelli, Massimiliano Bugiani, Lucio Casali, Marcello Ferrari, Gabriele Nicolini, Maria Grazia Panico, Pietro Pirina, Maria Elisabetta Zanolin, Isa Cerveri, Giuseppe Verlato

**Affiliations:** 1 Unit of Epidemiology and Medical Statistics, University of Verona, Verona, Italy; 2 Allergy Unit, Dept of Immuno-Allergic and Respiratory Diseases, Ospedali Riuniti di Ancona, Ancona, Italy; 3 Unit of Respiratory Medicine, ASL TO-2, Torino, Italy; 4 Unit of Respiratory Diseases, Dept of Internal Medicine, University of Perugia, Perugia, Italy; 5 Unit of Internal Medicine, University of Verona, Verona, Italy; 6 Medical Affairs Department, Chiesi Farmaceutici, Parma, Italy; 7 Epidemiology Unit, ASL SA-2, Salerno, Italy; 8 Institute for Respiratory Diseases, University of Sassari, Sassari, Italy; 9 Unit of Respiratory Diseases, IRCCS Policlinico San Matteo, University of Pavia, Pavia, Italy; Cardiff University, United Kingdom

## Abstract

**Background:**

The joint distribution of asthma and chronic obstructive pulmonary disease (COPD) **has** not been well described. This study aims at determining the prevalence of self-reported physician diagnoses of asthma, COPD and of the asthma-COPD overlap syndrome and to assess whether these conditions share a common set of risk factors.

**Methods:**

A screening questionnaire on respiratory symptoms, diagnoses and risk factors was administered by mail or phone to random samples of the general Italian population aged 20–44 (n = 5163) 45–64 (n = 2167) and 65–84 (n = 1030) in the frame of the multicentre Gene Environment Interactions in Respiratory Diseases (GEIRD) study.

**Results:**

A physician diagnosis of asthma or COPD (emphysema/chronic bronchitis/COPD) was reported by 13% and 21% of subjects aged <65 and 65–84 years respectively. Aging was associated with a marked decrease in the prevalence of diagnosed asthma (from 8.2% to 1.6%) and with a marked increase in the prevalence of diagnosed COPD (from 3.3% to 13.3%). The prevalence of the overlap of asthma and COPD was 1.6% (1.3%–2.0%), 2.1% (1.5%–2.8%) and 4.5% (3.2%–5.9%) in the 20–44, 45–64 and 65–84 age groups. Subjects with both asthma and COPD diagnoses were more likely to have respiratory symptoms, physical impairment, and to report hospital admissions compared to asthma or COPD alone (p<0.01). Age, sex, education and smoking showed different and sometimes opposite associations with the three conditions.

**Conclusion:**

Asthma and COPD are common in the general population, and they coexist in a substantial proportion of subjects. The asthma-COPD overlap syndrome represents an important clinical phenotype that deserves more medical attention and further research.

## Introduction

Asthma and chronic obstructive pulmonary disease (COPD) are a major public health problem because of their high and still rising prevalence, their associated morbidity, mortality and socio-economic costs [1]–[4].

Although asthma and COPD are different diseases, differential diagnosis is sometimes difficult and may be impossible in some older patients. [5] Furthermore, asthma and COPD may coexist: more than 40% of patients with COPD report a history of asthma, [6] and asthma has been recognized to be a risk factor for developing COPD. [7] Patients who have both COPD and asthma (overlap syndrome) have a more rapid disease progression, [8] a worse health-related quality of life, more frequent respiratory exacerbations, [9] increased co-morbidities and health care utilization than those with either disease alone [10]–[11].

The joint epidemiological distribution of asthma and COPD in the general population has not been thoroughly described. One reason is that the presence of the overlap syndrome is often an exclusion criterion in studies investigating either disease alone. [9], [12] Another reason is that studies on asthma are usually performed in populations of children or young adults, [13]–[14] where the prevalence of COPD is negligible, while studies on COPD are usually performed in elderly populations where the prevalence of asthma is low [15].

Assessing self-reported -physician-diagnosed -COPD and asthma in large representative samples of the general population is one of the simplest and most affordable methods to estimate the prevalence of these diseases. [16] Although the physician diagnosis has been questioned because of the potential inaccuracy, [17] recent studies showed that it has considerably improved during the last decade, probably due to increased lung function testing and dissemination of guidelines [18].

In this study we aimed at:

- determining the prevalence of the self-reported physician -diagnoses of asthma, COPD and of the asthma-COPD overlap syndrome in representative samples of young, middle-aged and elderly subjects from the general population in Italy;
- assessing whether subjects with asthma, COPD and the overlap syndrome diagnoses share common risk factors, and whether these vary with age.

For these purposes the data from the Gene Environment Interaction in Respiratory Diseases (GEIRD) study were used.

## Methods

### Study Design

GEIRD is a two-stage multicentre study started in 2007. [19] As a part of GEIRD stage 1, samples of about 3000 subjects aged 20–44 years (male : female = 1∶1) were randomly selected from the registry of the local health authority in four Italian centres: Turin, Pavia, Verona and Sassari. Additional random samples of about 1000 subjects aged 45–64 and 65–84 years were selected in 4 and in 2 centres respectively by the same procedure (**table 1**). All the eligible subjects were administered a postal screening questionnaire up to three times in the case of non response. A final phone interview was carried out to reach the remaining non responders.

**Table 1:** Number of responders (response rate %) by age-class in the four centres participating in the GEIRD stage 1 (screening questionnaire).

Centre	Age class (years)		
	[20–44]	[45–64]	[65–84]
Verona	1746 (67.7%)	676 (70.1%)	591 (60.1%)
Pavia	966 (37.1%)	460 (54.9%)	
Torino	1206 (54.7%)	502 (60.2%)	
Sassari	1245 (53.0%)	529 (62.8%)	439 (44.3%)
**Overall**	**5163 (53.0%)**	**2167 (62.3%)**	**1030 (52.2%)**

### Institutional Board Ethic Committee

Ethical approval was obtained in each centre from the appropriate institutional review board (***Turin***: Azienda Sanitaria Locale TO-2; ***Pavia***: “Istituto Ricovero e Cura a Carattere Scientifico”, Policlinico San Matteo; ***Verona***: “Istituti Ospedalieri di Verona”; ***Sassari***: “Azienda Ospedaliera Universitaria di Sassari”). All the participants were fully informed about all the aspects of the research project and consented to complete and return the questionnaire.

### Screening Questionnaire, Respiratory Outcomes and Potential Confounders

The GEIRD Screening Questionnaire (available on www.geird.org) is a modified version of questionnaires used in previous international and national studies. [20] ^.^ It is aimed at investigating the presence of symptoms of asthma, allergic rhinitis, chronic bronchitis/COPD and dyspnoea, and some environmental exposures. Questions on doctor diagnosis of asthma and COPD were also included.

Based on the answers to the questionnaire, a subject was considered to have a physician diagnosis of:

- *asthma* if s/he answered affirmatively to both questions “Have you ever had asthma?” and “Was this confirmed by a doctor?”;
- *COPD* if s/he gave a positive answer to the question: “Have you ever been told by a doctor that you have or had chronic bronchitis, chronic obstructive pulmonary disease (COPD) or emphysema?”.

The self reported physician diagnosis of COPD relied on the knowledge of the terms COPD, chronic bronchitis and emphysema. These are the most widely used terms when Italian doctors give patients a diagnosis of COPD.

The questionnaire also collected information on the presence of the following respiratory symptoms/conditions: wheezing or whistling in the chest in the last 12 months, asthma attacks in the last 12 months, current use of medicines for asthma, allergic rhinitis, chronic bronchitis (cough or phlegm on most days for a minimum of 3 months a year for at least 2 successive years). The dyspnoea scale of Medical Research Council (MRC) was used as a measure of the functional limitation due to breathlessness (grade ≥3: “do you get short of breath walking with other people of your own age on level ground, or do you have to stop for short of breath when walking at own pace on level ground?”) [21].

Gender, age, season when the questionnaire was filled in, type of contact (postal waves and telephone interview) were considered as potential confounders. Moreover, as the centres had different final response rates, the centre-specific cumulative response percentile rank to which subjects had answered was included in the analysis. [3] The following potential risk factors for respiratory symptoms were also included: smoking habits (current smoker, lifetime non-smoker and ex-smoker, defined as at least 1-year since quit), educational level (compulsory, high school, college/university education), the presence of industrial plants near home, self-reported heavy traffic near home (occasional/none, frequent, constant).

### Statistical Analysis

Categorical data were summarized as counts with percentages. Comparisons of variables across strata were performed by the Pearson Chi-squared test.

Age-sex adjusted prevalence rates of physician-diagnosed asthma and/or COPD were obtained through a logistic regression model with a dummy indicator of diagnosed asthma and/or COPD as dependent variables and centre, percentile rank of cumulative response, type of contact and season as covariates.

To study the joint distribution of asthma and COPD, a four-level indicator was obtained (0: neither asthma nor COPD; 1: asthma only; 2: asthma and COPD; 3: COPD only). The association of the previous conditions with potential risk factors was studied by fitting a multinomial regression model to the data, using the four-level indicator as the dependent variable, the other potential confounders and risk factors as independent variables. The interaction of age with other independent variables in determining the joint distribution of physician diagnoses of asthma and COPD was tested by likelihood-ratio test including in the regression model an appropriate interaction term.

Statistical analyses were performed with STATA 12.1 (Stata Corp LP, College Station, TX, USA).

## Results

### Response Rates and Sample Characteristics

The response rate (table 1) was minimum in the elderly (52.2%) and maximum in people aged 45–64 (62.3%). The distribution of sex and smoking habits showed a statistically significant variation (p<0.001) across age groups. The percentage of women was 53.6%, 52.2% and 42.7% in the 20–44, 45–64 and 65–84 yrs age groups, respectively. In these age groups, 26.6%, 23.4% and 9.6% of the subjects were current smokers, while 55.4%, 42.2% and 53.0% of the subjects never smoked.

### Prevalence of Self Reported Doctor Diagnosed Asthma and COPD

**Figure 1:**
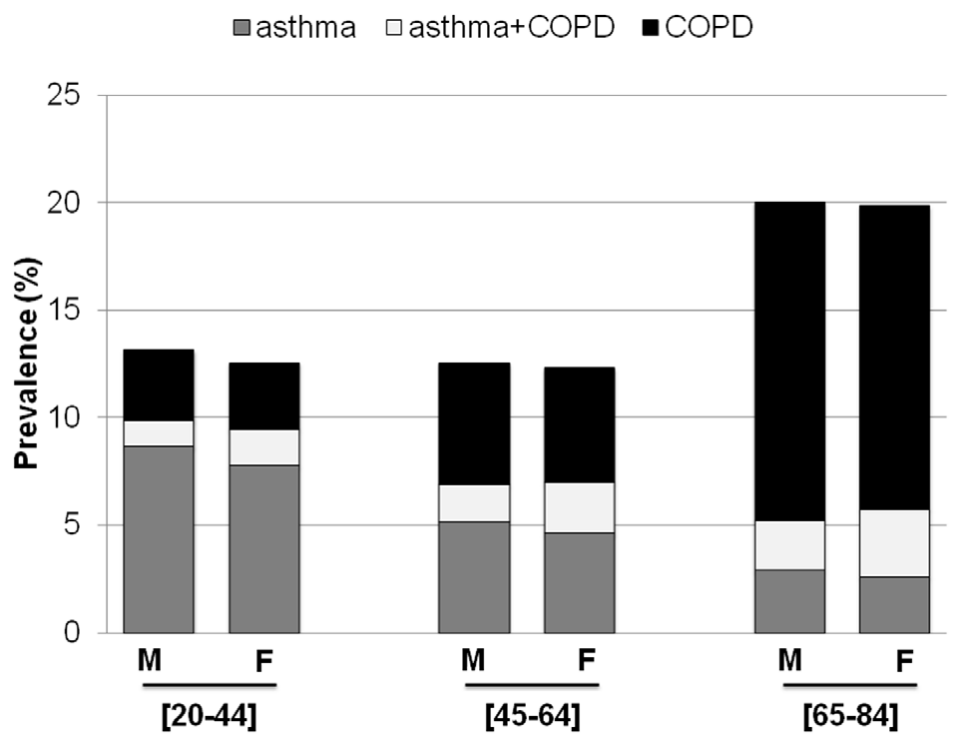
Prevalence* of self-reported physician-diagnosed asthma and/or COPD in the Italian population. *Adjusted for season, cumulative percentile rank of response, type of survey (postal waves/telephone) and centre.

The percentage of subjects with either the doctor diagnosis of asthma or COPD (**figure 1**) was almost constant up to 65 years of age: 13.1% (95%CI: 12.2%–14%) and 12.7% (95%CI: 11.3%–14.2%) in the 20–44 and 45–64 age classes respectively, and it increased to 20.7% (95%CI: 18.1%–23.3%) in the 65–84 age group, being similar in men and women.

The prevalence of the diagnosis of asthma alone almost halved (from 8.2% to 2.9%), while that of COPD alone almost doubled (from 3.3% to 13.3%) every twenty years of age (**table 2** and **figure 1**). The prevalence of asthma-COPD overlap syndrome significantly increased with age (p<0.001): it was 1.6%, 2.1% and 4.5% in the 20–44, 45–64 and 65–84 age groups, respectively.

**Table 2:** Joint distribution of self-reported doctor-diagnosed asthma and COPD.

Age class	Asthma only %(95%CI)	Asthma+COPD%(95%CI)	COPD only%(95%CI)
**[20–44]**	8.2 (7.5–9)	1.6 (1.3–2)	3.3 (2.8–3.8)
**[45–64]**	4.9 (4–5.9)	2.1 (1.5–2.8)	5.7 (4.7–6.7)
**[65–84]**	2.9 (1.8–4)	4.5 (3.2–5.9)	13.3 (11.1–15.5)

Prevalence (%) with 95% confidence interval (CI).

Among subjects who reported physician-diagnosed asthma, the percentage of the asthma-COPD overlap syndrome was 16%, 30% and 61% in the 20–44, 45–64 and 65–84 age groups, respectively. Conversely, among subjects who reported COPD the percentage of the asthma-COPD overlap syndrome was 33%, 27% and 25% in the three age group, respectively.

### Distribution of Symptoms, Physical Limitation and Hospitalization According to the Diagnoses of Asthma and COPD

Respiratory symptoms, physical limitation (MRC≥3) and hospitalization were statistically significantly increased in subjects with either doctor diagnosed asthma or COPD or both (p<0.001) The risk of having respiratory symptoms or using medicines ranged from a minimum of 5-fold (wheezing) to a maximum of 200-fold (current use of anti-asthmatic drugs) with respect to subjects without a doctor diagnosis (**table 3**). Subjects reporting the diagnosis of the asthma-COPD overlap syndrome had the highest prevalence of all the respiratory symptoms/conditions considered, with the exception of allergic rhinitis and they differed significantly from the subjects with the diagnosis of asthma or COPD alone (see the non overlapping of confidence intervals in table 4) for the majority of symptoms investigated.

**Table 3:** Prevalence* (% with 95%CI) of respiratory symptoms or conditions in subjects who did and did not report a diagnosis of asthma and/or COPD.

Respiratory symptoms or conditions	no asthma, no COPD %(95%CI)	asthma only %(95%CI)	asthma+COPD overlap %(95%CI)	COPD only %(95%CI)
Wheezing	9.9 (9.2–10.6)	43.4 (39.2–47.7)	78.7 (71.3–84.5)	42.7 (37.6–47.9)
Asthma attacks	0.7 (0.5–0.9)	38.8 (34.6–43.2)	56.9 (48.7–64.8)	4.4 (2.7–6.9)
Antiasthmatic drugs	0.3 (0.2–0.4)	29.8 (25.8–34)	55.4 (47–63.5)	2 (1.1–3.8)
Allergic rhinitis	18.2 (17.3–19.1)	59.2 (54.9–63.4)	53.5 (45.5–61.3)	23.9 (19.7–28.6)
Cough or phlegm	10.2 (9.5–10.9)	23.1 (19.6–26.9)	61.7 (53.7–69.1)	54 (48.7–59.2)
MRC† ≥3	3.8 (3.3–4.3)	9.3 (7.1–12.2)	38.8 (31.1–47.1)	20.8 (17–25.2)
Hospitalizations	0.4 (0.2–0.5)	1.1 (0.5–2.4)	3.1 (1.4–6.7)	2.5 (1.4–4.5)

* Adjusted for gender, age (class), season, % of answers to the questionnaire, type of survey (postal/telephone), and centre.

† MRC: Medical Research Council dyspnea score.

**Table 4:** Factors associated with the diagnoses of asthma, COPD, and the asthma–COPD overlap syndrome.

	Asthma only	Asthma+COPD	COPD only
**Gender** males	1	1	1
females	0.87 (0.72–1.04)	**1.63 (1.15–2.31)**	1.15 (0.92–1.44)
**Age** [20–44]	1	1	1
[45–64]	**0.60 (0.47–0.77)**	1.12 (0.75–1.69)	**1.57 (1.20–2.07)**
[65–85]	**0.48 (0.3–0.77)**	1.43 (0.76–2.70)	**5.50 (3.75–8.08)**
**Smoking habits** non-smoker	1	1	1
current smoker	**1.27 (1.02–1.58)**	**1.70 (1.12–2.60)**	**3.16 (2.41–4.16)**
ex smoker	0.99 (0.78–1.26)	**1.56 (1.04–2.35)**	**1.56 (1.16–2.08)**
**Education** compulsory	1	1	1
high school	1.26 (0.99–1.6)	**0.45 (0.31–0.65)**	**0.70 (0.55–0.90)**
college/university	**1.61 (1.24–2.09)**	**0.30 (0.17–0.51)**	**0.38 (0.26–0.57)**
**Industries nearby**	1.00 (0.78–1.27)	0.91 (0.55–1.50)	1.30 (0.98–1.73)
**Heavy traffic** occasional/none	1	1	1
frequent	1.00 (0.78–1.27)	0.91 (0.55–1.50)	**1.30 (0.98–1.73)**
constant	**1.38 (1.07–1.77)**	1.57 (0.98–2.50)	**1.67 (1.24–2.24)**

Relative Risk Ratios* (RRR) with 95%CI, with subjects without diagnosed diseases as reference group. Statistically significant associations are shown in bold.

* adjusted for all variables presented in this table plus design confounders (season, cumulative percentile rank of response, type of interview, GEIRD centre).

### Factors Associated with the Diagnosis of Asthma and COPD

Women were more likely to have the asthma-COPD overlap syndrome compared to men (RR = 1.63; 95%CI: 1.15–2.31). The risk of reporting a diagnosis of asthma only was significantly higher in young (p = 0.001) and highly educated people (p = .002), while the opposite held true for COPD, with or without asthma (**table 4**) (p<.001).

Smoking was significantly associated with both asthma and COPD. Ex-smokers were at a lower risk compared to current smokers, but they had a significantly higher risk of COPD (RR = 1.56; 95%CI:1.16–2.08) and of the COPD-asthma overlap syndrome (RR = 1.56; 95%CI: 1.04–2.35) with respect to non-smokers. Higher levels of heavy traffic near home were significantly associated with a greater risk of having diagnosed COPD. Age did not modify the associations of these risk factors with the diagnoses of asthma, COPD or both.

## Discussion

The main findings of this analysis are:

- About 1 out of 8 subjects <65 yrs old and 1 out of 5 subjects aged 65 or more yrs reported a physician diagnosis of asthma or COPD. Aging was associated with a marked decrease in the prevalence of asthma and with a marked increase in the prevalence of COPD. However, the percentage of subjects reporting either diagnosed asthma or COPD or both was almost constant until 65 years of age;
- The prevalence of the self-reported physician-diagnosed asthma-COPD “overlap syndrome” in Italy ranged from 1.6% in the 20–44 age class to 4.5% in the 65–84 age class. Subjects with the overlap syndrome had a statistically significantly higher frequency of respiratory symptoms, functional limitation and hospitalization with respect to subjects with the diagnosis of asthma or COPD alone;
- Age, sex, education and active smoking showed different and sometimes opposite associations with asthma, COPD and the overlap syndrome. Age did not modify the associations of the previous factors with respiratory diseases.

### Prevalence of Asthma and COPD in the General Population Aged 20–85 Years

There is a paucity of knowledge on the prevalence of asthma in the elderly, [22] probably because asthma and COPD tend to overlap, making the diagnosis complex. [23], [24] On the contrary, the large majority of epidemiological studies on COPD, which is usually assumed to be an “aging disease”, has been focused on elderly population and only few studies have been performed on young adult populations [25].

Our study is one of the few that reports the prevalence of both diseases in people aged from 20 to 84 years. It documents that asthma and COPD are major health problems, affecting about 13% of adults and 20% of the elderly. As people got older, the prevalence of asthma decreased (from 8.2% to 2.9%), while the prevalence COPD increased (from 3.3% to 13.3%). However, the prevalence of asthma and COPD remained non negligible even at the extremes of the age range. It is likely that this age-related pattern of asthma and COPD reflects both the true pattern of disease prevalence and the differential doctor’s diagnostic propensity according to the age of their patients (diagnostic bias). [23] Indeed, distinguishing between asthma and COPD can be quite challenging, even for the most expert medical professional, and COPD is often misdiagnosed as asthma in young people, while the opposite happens in the elderly. [26] This could explain in part our finding showing that the prevalence of asthma and/or COPD is invariant in the 20–65 age range.

Our estimates of the prevalence of asthma in Italy based on the self-reported doctor diagnosis are in line with those recently obtained on adult representative national samples, by using both an internationally validated questionnaire and the diagnosis of asthma made by Italian General Practitioners. [3], [27] The agreement between prevalence estimates from health interview surveys and those from GP registration may indicate an improvement in the ability of general practitioners to diagnose asthma during the last decade, which is probably due to the increased diffusion of spirometry and dissemination of asthma guidelines [18].

At variance, the prevalence estimates of COPD may change considerably according to the diagnostic tools used. In general, epidemiological studies based on physician diagnoses reported lower prevalence estimates than those relying on spirometry, [15], [28] which is the gold standard definition for COPD (GOLD guidelines). [2] As a consequence of the definition of COPD used in our study, a certain degree of misclassification probably occurred: some subjects with spirometrically-defined COPD might not have had a doctor diagnosis, and a non-negligible percentage of subjects who reported a physician-diagnosis might not have met the spirometric criteria. [29] Despite its limitation, the physician-diagnosed COPD continues to be widely used in epidemiological surveys on large representative national samples, [16], [30] either because of its simplicity and cost saving, and because it gives us a “real life” picture of the percentage of people in a country that are diagnosed and treated as COPD patients by doctors. Our estimates of the prevalence of physician-diagnosed COPD in Italy are in the range of those reported in other countries [24], [31].

### Prevalence of the Asthma-COPD Overlap Syndrome in the General Population

It is well known that some patients suffer from both asthma and COPD, and that they represent an important clinical population with peculiar characteristics. [6], [29] In fact, it has been frequently observed that some patients with asthma have an accelerated decline in lung function, especially if they are smokers, [8], [32] and that some patients with COPD have a good response to treatment with bronchodilators and ICS. It is still an open question whether the overlap syndrome represents the coexistence of two distinct airway diseases or whether there are common underlying pathogenic mechanisms leading to this phenotype. [33] In comparison to previous studies that have considered selected groups of patients, such as COPD patients, [11], [34] our study enabled us to assess the prevalence of the asthma-COPD overlap syndrome in the general population. We found that this prevalence ranged from a minimum of 1.6% (95%CI: 1.3%–2.0%) in the 20–44 age group to 4.5% (95%CI: 3.2%–5.9) in the 60–84 age group.

These data point out that the asthma-COPD overlap syndrome affects many subjects and becomes more prevalent with advancing age. Furthermore, in agreement with previous studies, [9]–[11] we found that patients with the overlap syndrome were more likely to have respiratory symptoms, physical impairment and to report hospital admission for respiratory diseases compared to subjects with asthma or COPD alone. In summary, our findings clearly illustrate that there are many reasons to focus medical attention and increase the research efforts on asthma-COPD overlap syndrome patients.

### Risk Factors of Asthma, COPD and of the Overlap Syndrome

Asthma-COPD overlap syndrome can develop when there is an accelerated decline in lung function, or incomplete lung growth, or both. [23], [24] The determinants for these events, like tobacco smoke exposure *etc*., are presumed to be shared with asthma and COPD. [32], [35] However, the distribution of risk factors across asthma, COPD and the overlap syndrome has not been thoroughly investigated and may offer new insights into the mechanisms of the different respiratory diseases.

Our findings show that the risk factor profiles of subjects with the diagnosis of asthma, COPD and asthma-COPD overlap syndrome are different, even if they share some common patterns. Women had a higher susceptibility for the asthma-COPD overlap syndrome (OR:1.63; 95%CI: 1.15–2.31) than men, while gender was not associated with either the prevalence of asthma or COPD alone. Accordingly, the higher prevalence of adult asthma reported in women could be at least partially due to their increased susceptibility to the overlap syndrome [36].

In agreement with a previous practice-based study performed by the Italian College of General Practitioners, [27] age was strongly and negatively associated with the prevalence of asthma alone, while the opposite occurred for the prevalence of COPD alone. The prevalence of the overlap syndrome showed only a weak positive association with aging, after adjusting for the other potential confounders. Considering that the incidence of asthma has been constantly on the increase during the last century, the negative association of the prevalence of asthma with age might be due, at least in part, to a “generational” effect [3] as well as to the doctors’ diagnostic propensity.

Although it is recognised that active and passive smoking are the major risk factors for COPD, [2] there are conflicting results on the association between asthma and smoking. [37] In our study, smoking was significantly and positively associated with all the three diseases, however the strength of the association was much higher for COPD than for asthma or the overlap syndrome.

Differently from some studies, [38] and in agreement with others, [39] we found that more educated people had significantly higher prevalence rates of asthma. Apparently contradictory evidence regarding the relationship between social class and asthma could be related to differences in the in the geographical area studied or in the definition of asthma. [40].

In agreement with a large body of epidemiological evidence, [2] our data show that the prevalence of COPD is significantly lower in more educated people. A similar association was found for the asthma-COPD overlap syndrome. The presence of the overlap syndrome among asthmatics could therefore explain why other studies found an inverse association between asthma and education.

As previously reported, [41] we found a clear and statistically significant association between self-reported exposure to traffic air pollution and the prevalence of COPD. The prevalence of asthma and of the asthma-COPD overlap syndrome was definitely less influenced by the exposure to air pollution.

In summary, the heterogeneity of the associations of the studied risk factors across the different respiratory diseases shown in our analysis seems to suggest that asthma, COPD and the asthma-COPD overlap syndrome could represent different phenotypes.

### Limits of the Study

To our knowledge, this study provides the first large scale estimate of the joint distribution of asthma and COPD in general population. It has a number of limitations. Firstly, it was not possible to assess if and how asthma and COPD interact in the pathogenesis of the overlap-syndrome and which of the two occurred first, because of the cross-sectional design of the study. Then, our definitions of asthma and COPD were based on self-reported physician-diagnoses of the disease, and did not rely on lung function tests. As such, there might be an issue of recall bias. However, these definitions have been widely used in large international surveys, [13]–[14] and have proved to be reasonably reliable. [15] Our definition of COPD was based on the physician diagnosis of at least one of three conditions (COPD, emphysema and chronic bronchitis), and this might have resulted in an overestimation of the actual prevalence of COPD. This definition was used because, for the sake of simplicity, Italian GPs often use the terms “emphysema” and “chronic bronchitis” when giving a COPD diagnosis to their patients. Furthermore it is also likely that our definition of diseases based on the doctor diagnoses could have led to an underdiagnosis of a certain proportion of the population, as suggested by the fact that about 10% and 1% of the subjects without a diagnosis of asthma or COPD reported wheezing and asthma attacks in the last year respectively. Furthermore the relatively low number of elderly subjects included in our study (subjects aged 65 yrs or more were recruited in only 2 out of 4 centers) and the predominant white European population of Italy may partially limit the generalizability of our results.

The overall response rate was quite good with an average of over 50% in all age groups. The main outlier was the centre of Pavia, with a significantly lower response rate in the 20–44 age group. However, the distribution of asthma and COPD in the centre of Pavia was similar to those obtained in the other centers (data not reported), suggesting that the low response rate in this centre did not bias our results.

### Conclusion

Asthma and COPD affect more than one out of eight of adults aged 20 years or older. The coexistence of both diseases is present in a substantial proportion of subjects and increases with advancing age. Subjects with the asthma-COPD overlap syndrome seem to be a specific phenotype that has more respiratory symptoms, more physical impairment, consume more medical resources and have a peculiar pattern of risk factors compared to asthma or COPD alone. Improvements in monitoring, in the treatment and in the research on the asthma-COPD overlap syndrome are necessary.
